# Direct Parental (DIPA) CRISPR in the jewel wasp, *Nasonia vitripennis*

**DOI:** 10.1093/g3journal/jkae095

**Published:** 2024-05-12

**Authors:** Xinmi Zhang, Anabhra Singh, Kassandra Soriano Martinez, Patrick M Ferree

**Affiliations:** Department of Natural Sciences, Pitzer College and Scripps College, 925 N Mills Ave, Claremont, CA 91711, USA; Department of Natural Sciences, Pitzer College and Scripps College, 925 N Mills Ave, Claremont, CA 91711, USA; Department of Natural Sciences, Pitzer College and Scripps College, 925 N Mills Ave, Claremont, CA 91711, USA; Department of Natural Sciences, Pitzer College and Scripps College, 925 N Mills Ave, Claremont, CA 91711, USA

**Keywords:** *Nasonia vitripennis*, DIPA, gene editing, microinjection, vitellogenesis

## Abstract

While clustered regularly interspaced short palindromic repeats (CRISPR)–Cas9 technology has demonstrated remarkable promise as a gene-editing tool, its application in certain insects, such as the jewel wasp, *Nasonia vitripennis*, has been hindered by a lack of a tractable method for reagent delivery. Direct Parental (DIPA-) CRISPR recently emerged as a facile way to induce gene lesions because it involves adult injection with commercially available Cas9–sgRNA with no helper reagent. However, DIPA-CRISPR has so far been tested in only a few insects. Here, we have assessed the amenability of DIPA-CRISPR in *N. vitripennis* by targeting two eye pigmentation genes, *cinnabar* and *vermilion*, which function in the ommochrome pathway. Successful generation of lesions in both genes demonstrated the functionality of DIPA-CRISPR in *N. vitripennis* and its potential application to other genes, thereby expanding the range of insects suitable for this method. We varied two parameters, Cas9–sgRNA concentration and injection volume, to determine optimal injection conditions. We found that the larger injection volume coupled with either higher or lower reagent concentration was needed for consistent mutation production. However, DIPA-CRISPR yields an overall low mutation rate in *N. vitripennis* when compared to other tested insects, a characteristic that may be attributed to a proportionally low vitellogenic import efficiency in the jewel wasp. We discuss different factors that may be considered in determining when DIPA-CRISPR may be preferable over other reagent delivery methods.

## Introduction

Clustered regularly interspaced short palindromic repeats (CRISPR)–Cas9 technology, has proved to be effective across a wide range of organisms for precise alteration of target genes. Traditionally, CRISPR involves the direct delivery of Cas9–sgRNA ribonucleoprotein (RNP) particles via microinjection into preblastoderm embryos (Gratz *et al.* 2013; Wang *et al.* 2013; Dong *et al.* 2015). Imperfect repair of targeted lesions by nonhomologous end joining in the nuclei destined to become germline stem cells will result in mutant alleles that can be transmitted to progeny. CRISPR has been well-established in traditional genetic model organisms like *Drosophila melanogaster*, in which transgenic lines that express Cas9 and sgRNA have been produced (Kondo and Ueda 2013; Sebo *et al.* 2014), thus bypassing the need for embryonic microinjection. While such approaches have accelerated CRISPR applications in *D. melanogaster*, they are lacking in other nonmodel organisms that are nevertheless used frequently for experimentation. The use of CRISPR is, barring unusual circumstances, expected to be universal across eukaryotes (Hillary *et al.* 2020). Indeed, CRISPR-based mutagenesis, such as via direct injection of reagents into embryos, has been demonstrated to work successfully in a growing number of model and nonmodel organisms (Taning *et al.* 2017; Singh *et al.* 2022). However, this approach can be challenging in the case of very small and fragile embryos, a limited number of available embryos, or when obtaining embryos is difficult, such as in viviparous organisms.

To circumvent these barriers of embryo microinjection, a CRISPR-based method known as Receptor-Mediated Ovary Transduction of cargo (ReMOT) was developed as an alternative approach (Chaverra-Rodriguez *et al.* 2018). ReMOT utilizes a modified Cas9 protein fused with a peptide ligand called P2C, which is derived from the *D. melanogaster* yolk protein precursor (Chaverra-Rodriguez *et al.* 2018). P2C–Cas9 and sgRNAs are combined and co-injected into adult females, and the RNP particles are imported into the oocytes of egg chambers undergoing vitellogenesis (Terradas *et al.* 2023). This approach was found to yield a G0 gene-editing efficiency (GEF: The proportion of edited G0 individuals out of the total number of G0 s) of ∼1.5% in the mosquito *Ades aegypti* (Chaverra-Rodriguez *et al.* 2018). However, this method was reported to be less efficient in other insects, including the jewel wasp, *Nasonia vitripennis* (GEF = 0.24%) and the red flour beetle, *Tribolium castaneum* (GEF = 0.26%) (Chaverra-Rodriguez *et al.* 2020; Shirai and Daimon 2020). Moreover, conducting this method requires the use of purified P2C–Cas9, preferably using a yolk protein precursor ligand that is derived from the specific experimental organism being employed (Shirai and Daimon 2020).

Recently, a similar approach known as Direct Parental (DIPA-) CRISPR has shown strong promise as a simpler, more tractable way of generating edited genes in several rising model insects, including the cockroach, the red flour beetle, and the yellow fever mosquito (Shirai *et al.* 2022, 2023). Like the ReMOT approach, DIPA-CRISPR involves the injection of Cas9–sgRNA RNP particles into adult females. However, DIPA-CRISPR does not utilize P2C–Cas9, but instead the unaltered form of Cas9 (Shirai *et al.* 2022). Impressively, DIPA-CRISPR has yielded mutation frequencies with GEF values as high as 21.8% in the German cockroach *Blattella germanica* and 71.4% in *T. castaneum* (Shirai *et al.* 2022). The lack of need for a purified, species-specific P2C-tagged Cas9 makes this approach feasible for laboratories lacking such specialized resources. Additionally, it was demonstrated that insertion of small ssDNA into targeted sites via homology-directed repair is achievable through DIPA-CRISPR in cockroaches (Shirai *et al.* 2022). An important question is whether DIPA-CRISPR is as effective a method for generating gene edits in other rising model insects in addition to those examined thus far (Shirai *et al.* 2022, 2023).

Expanding such feasible CRISPR-based approaches to a wider range of insects will significantly facilitate gene-level studies in these organisms. One such group consists of *N. vitripennis* and its sibling species, *N. longicornis* and *N. giraulti*. These small, chalcid wasps are natural parasitoids of carrion flies such as flesh flies and blow flies (Whiting 1967; Werren and Loehlin 2009). Among the *Nasonia* species, *N. vitripennis* is the most extensively studied and commonly used for laboratory experimentation (Whiting 1967; Werren and Loehlin 2009). Due to its advantages of easy rearing, a short generation time, and a high-quality, fully sequenced genome (Werren and Loehlin 2009), *N. vitripennis* has become a rising model organism for studies of comparative genomics, development, physiology, and behavioral ecology (Werren and Loehlin 2009). Like all hymenopteran insects (including all wasps, as well as bees, ants, and sawflies), *N. vitripennis* exhibits haplo-diploidy, a reproductive system in which females develop as diploids from fertilized eggs, while males develop as haploids from unfertilized eggs (Rasplus *et al.* 2010). This distinctive system facilitates genetic analysis because phenotypic effects of strong loss-of-function mutations are clearly revealed in haploid males, and such mutations can be easily maintained in heterozygous females (Werren *et al.* 2010). Additionally, the parasitoid behavior of *N. vitripennis* has made this insect a promising agent for the biological control of pest fly species (Danneels *et al.* 2010).

Prior to the development of CRISPR–Cas9 technology, there were limited tools available for the manipulation of gene function in *N. vitripennis*. For example, systemic RNA interference (sRNAi) can be used to suppress gene function either in the injected individual itself or parentally, i.e. in the progeny of injected individuals (Lynch and Desplan 2006; Dalla Benetta et al. 2020a; Zou *et al.* 2020). However, a drawback of sRNAi is that its phenotypic effects are transient, necessitating iterative sRNAi treatments for long-term studies of gene function using this method. Thus, the development of user-friendly, CRISPR-based methods to create stable, heritable mutant alleles in *N. vitripennis* would be advantageous. Microinjection of CRISPR reagents into early embryos has been successfully used to generate mutant alleles of the eye pigmentation gene, *cinnabar* (*cn*), in *N. vitripennis* (Li *et al.* 2017). Subsequently, mutations in this same gene were achieved using ReMOT (Chaverra-Rodriguez *et al.* 2020). For reasons previously mentioned, these approaches have drawbacks, thus hindering the use of *Nasonia* in reverse-genetic studies.

In this study, we explored the potential of DIPA-CRISPR as a more amenable way of site-directed mutagenesis in *N. vitripennis*. Our results reveal that the mutation frequency generated by DIPA-CRISPR in *N. vitripennis* is substantially lower than frequencies achieved in the cockroach and the red flour beetle (Shirai *et al.* 2022), and moderately lower than the ReMOT approach in *N. vitripennis* (Chaverra-Rodriguez *et al.* 2020). Nevertheless, we propose that this method can be successfully used for certain types of functional gene studies in this insect. We provide a detailed description of our methodological approach, which holds potential for expansion to other experimental goals.

## Materials and methods

### Insect maintenance

The *AsymCx* strain of *N. vitripennis* (Werren and Loehlin 2009) was used for CRISPR-directed mutagenesis in this study. The maintenance of wasps was conducted by following a procedure established by Werren and Loehlin (2009). Specifically, ∼5 females and the same number of males were allowed to mate in a 12 × 75 mm vial, and 4−5 *Sarcophaga bullata* pupae (Ward's Science, USA) were provided as hosts. The vials were kept at 25°C in a 12:12 h light-to-dark photoperiod. Before adult emergence, female wasp pupae were sorted from their host puparia and placed into a separate vial. Following emergence of the adult females, a small droplet of 1:1 honey in water was applied to the inside of the vial for feeding and hydration.

### CRISPR target genes

**Fig. 1.**
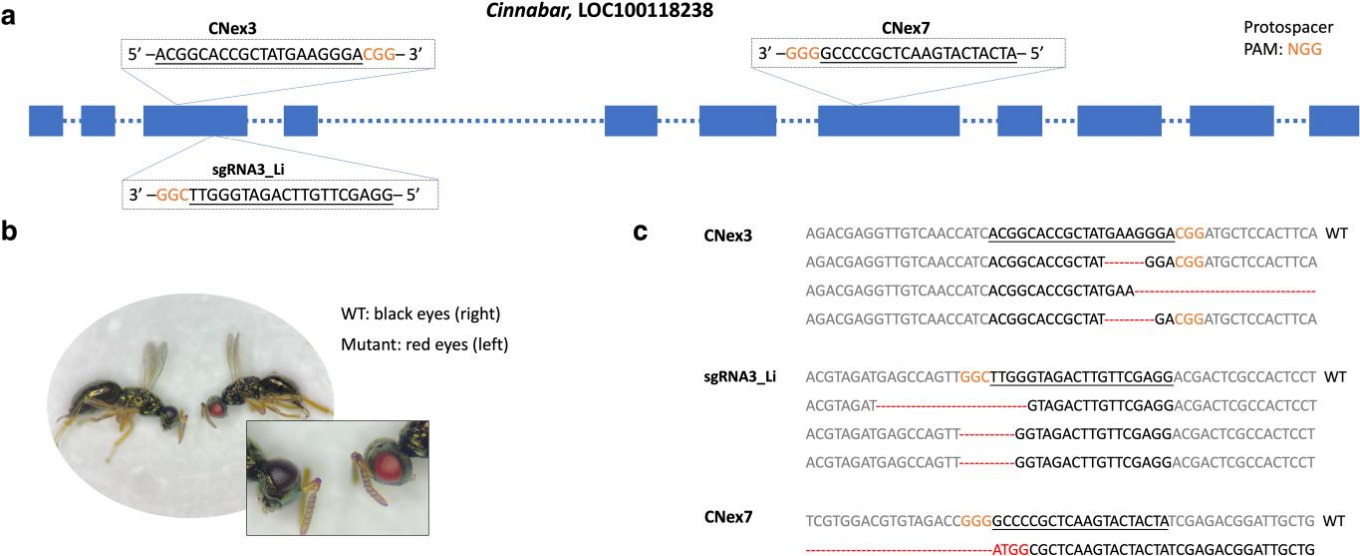
CRISPR/Cas9 target sites in the *cn* gene (a), a representative mutant phenotype (b), and selected lesions produced in the study (c). a) Blue boxes represent exons; dashed lines represent introns; sgRNAs with the protospacer-adjacent motif (PAM) sequences (NGG), are indicated in the box located within the corresponding exons; the sgRNAs are underlined and the PAM sequences are highlighted in orange. b) Mutant and WT G0 males from DIPA-CRISPR-treated female wasps. c) Insertions and deletions are shown in red.

The *cn* gene (Accession number LOC100118238) was selected as an initial DIPA-CRISPR target (Li *et al.* 2017). *cn* encodes kynurenine 3-monooxygenase, an enzyme functioning in the ommochrome pigmentation pathway, which is responsible for insect eye color (Linzen 1974; Vargas-Lowman *et al.* 2019). Loss-of-function mutations in the *cn* gene result in a distinctive, red-eyed mutant phenotype in adult *N. vitripennis*, making this gene an ideal target using CRISPR. A total of three sgRNAs were employed for DIPA-CRISPR: One was sourced from Li *et al.* (2017) and the other two were newly designed using CHOPCHOP v3 (Labun *et al.* 2019) (see Fig. 1 for sequences and locations).

**Fig. 2.**
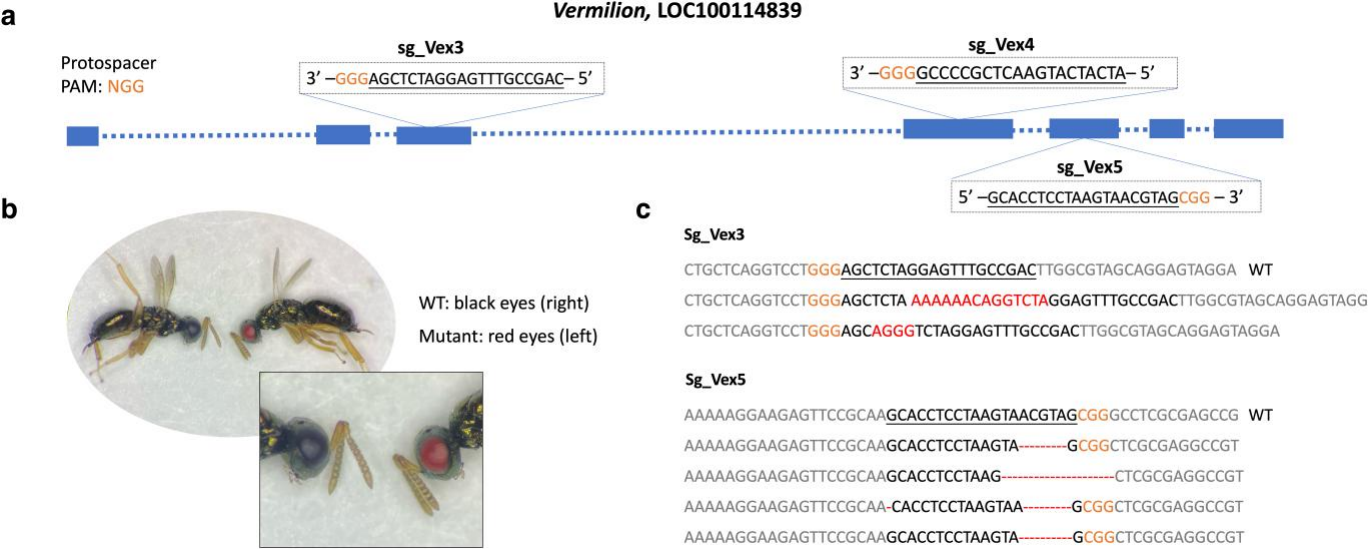
CRISPR/Cas9 target sites in the *v* gene (a), a representative mutant phenotype (b), and lesions that were induced in *v* (c). a) Blue boxes represent exons and dashed lines represent introns; sgRNAs and the PAM (NGG) sequence are indicated in the box located within the corresponding exons. The PAM sequence (NGG) is highlighted in orange. b) Representative mutant and WT G0 progeny produced by DIPA-CRISPR-treated female wasps. c) Insertions and deletions are shown in red.

The *vermilion* (*v*) gene was selected as an additional target. The *v* gene (Accession number LOC100114839) encodes tryptophan 2,3-dioxygenase, which functions upstream of the *cn* gene in the ommochrome pathway (Xu *et al.* 2020). Three sgRNAs (see Fig. 2 for sequences and locations) intended to target *v* were designed using CHOPCHOP v3 (Labun *et al.* 2019). To initially verify the phenotype resulting from the disruption of the *v* gene, sRNAi was conducted following the procedure outlined below.

### Preparation of double-stranded RNA for sRNAi

The mRNA sequence of the *v* gene was obtained from NCBI. Primers (Table 1: Nvv_R2_F&R) designed by using the NCBI primer-BLAST feature were used to amplify a 506 bp PCR product from wild type (WT) wasp cDNA. For cDNA preparation, total RNA was extracted from WT *N. vitripennis* adults using the RNeasy Mini Kit (QIAGEN, Germany). cDNA was synthesized using the RevertAid H Minus First Strand cDNA Synthesis Kit (ThermoFisher, USA).

**Table 1.** Primers information.

Primer	Sequence	Annealing temperature
Nvv_R2_F	5′—AGTACTTGCGCCTCGACAAA—3′	68°C (Q5)
Nvv_R2_R	5′—TTTGATCGCCTCAATGGCCT—3′	
T7_Nvv_R2_F	5′—TAATACGACTCACTATAGGGAGTACTTGCGCCTCGACAAA—3′	72°C (Q5)
T7_Nvv_R2_R	5′—TAATACGACTCACTATAGGGTTTGATCGCCTCAATGGCCT—3′	
Nvcn_F	5′—CCATGTCGAGCTTAAAAATCGACG—3′	66°C (Q5)
Nvcn_R	5′—CTCGTGTTGTATCATCGAAGCATC—3′	
qcnm_F	5′—CGAGAGTCGAGAAATCGTCA—3′	56°C (Taq)
qcnm_R	5′—CGAGGTCTCTTCGACATCAT—3′	
Ef1α_F	5′—CACTTGATCTACAAATGCGGTG—3′	56°C (Taq)
Ef1α_R	5′—CCTTCAGTTTGTCCAAGACC—3′	
Ak3_F	5′—AATTCAATCGGGTTCTGCTC—3′	56°C (Taq)
Ak3_R	5′—CAGCATCTCATCTAACTTCTCTG—3′	

The annealing temperature was calculated according to the specific DNA polymerase used, which is indicated in parentheses.

PCR amplification was conducted using Q5 high-fidelity 2X Master Mix (New England BioLabs Inc., USA). PCR conditions followed the instructions for Q5 high-fidelity DNA polymerase on the New England BioLabs website. After confirming the PCR product on an agarose gel, a new primer set in which the T7 promoter sequence was added to 5′ ends of both primers (Table 1: T7_Nvv_R2_F&R) was used to produce a transcribable version of the PCR product. This product was purified using QIAquick PCR Purification kit (QIAGEN, Germany) and used to synthesize double-stranded RNA (dsRNA). Bidirectional RNA synthesis was conducted using the MEGAscript RNAi kit following the manufacturer's protocol. The resulting dsRNA was ethanol-precipitated, partitioned into 5 μl aliquots, and stored at –80°C. Stocks of dsRNA were ∼2 μg/μl in concentration.

### Early pupal injection for sRNAi

For injection, the FemtoJet 4i microinjector (Eppendorf SE, Germany) was used. The injection pressure for controlling the reagent flow was set between 200 and 400 pi, depending on the opening of the needle. The injector's manual mode was used to control the time of injection.

dsRNA was diluted with nuclease-free water to a working concentration of ∼1 μg/μl. A light dilution (1 part dye to 6 parts nuclease-free water) of red food dye was added to the dsRNA to help visualize sufficient injection of each animal (a slight red discoloration can be seen through the semi-transparent pupal exoskeleton following successful injection). Wasps in the yellow or yellow-light-pink-eyed pupal stage were placed into a thin layer of clear Elmer's school glue on a glass slide, with the ventral side of the pupae facing upward. Further details of pupal injection have been previously described in Dalla Benetta *et al*. (2020b). Upon emergence of the injected wasps, the degree of eye pigmentation was compared to that of WT wasps to confirm *v* gene targeting.

### Cas9–sgRNA RNP preparation

Purified Cas9 protein was obtained from PNA BIO INC (USA), and sgRNAs were synthesized by Synthego (USA). Stocks of the Cas9 and sgRNAs were made by adding nuclease-free water to each reagent in dry form to produce 8 μg/μl of each; they were aliquoted into 1 μl aliquots and stored at –80°C for later use. To form an injectable RNP complex, Cas9 and sgRNAs were mixed and incubated at room temperature for 15 min. The high concentrations used for injection were 4 μg/μl of Cas9 and 1.3 μg/μl of each sgRNA; these concentrations are consistent with those employed in a previous study (Shirai *et al.* 2022, 2023). The lower concentration mixture was achieved by using reagents that were diluted to one-half of these concentrations. The RNP mixture was kept on ice throughout the injection process.

### Adult and late pupal injection for DIPA-CRISPR

Adult female wasps aged <2 days were used for CRISPR injections as previously described (Dalla Benetta *et al*. 2020b). Briefly, adult wasps were immobilized on the surface of an ice-cooled Petri dish. The wasps were carefully stabilized using the bristles of a fine paintbrush or the blunted tip of forceps, with their ventral side facing upward. The RNP reagents were injected into the abdomen of female wasps by inserting the needle underneath the 2nd or 3rd tergite. A large injection volume was inferred by an observable slow swelling of the abdomen until its full expansion, as well as expansion of other body parts such as wings and anus, was visible. The lower reagent volume was indicated by only a slight swelling of the abdomen.

Cas9–sgRNA injections were also performed on fully pigmented pupae (the stage immediately preceding eclosion) to assess editing efficiency at an earlier developmental time. Like pupal injection for sRNAi, RNP reagents were injected into the abdomen of pupae that were immobilized on a glass slide using glue with the ventral side facing upward. For injection into dark pupae, the needle must pass through two layers—the old exoskeleton and, just beneath, the new cuticle—to ensure the delivery of reagents inside the wasp. The midline of the abdomen for both pupal and adult wasps was carefully avoided during needle insertion to avoid damaging the female's ovipositor.

### Maintenance of Cas9–sgRNA injected animals

Injected adult females or emerged females that had been injected as pupae were kept individually in glass vials at room temperature (∼20–22°C) for a 1-day recovery period. On the second day postinjection, a host pupa was added to the vial and left for 2 days at room temperature. Subsequently, the host, containing developing G0 progeny (i.e. the progeny produced by the injected parent), was removed and placed into a separate vial. A second host pupa was then offered to the injected female for further oviposition. All hosts containing developing G0 progeny were maintained at 25°C with a 12: 12 h light-to-dark photoperiod.

### Phenotypic screening for mutants and subsequent genetic crosses

**Fig. 3.**
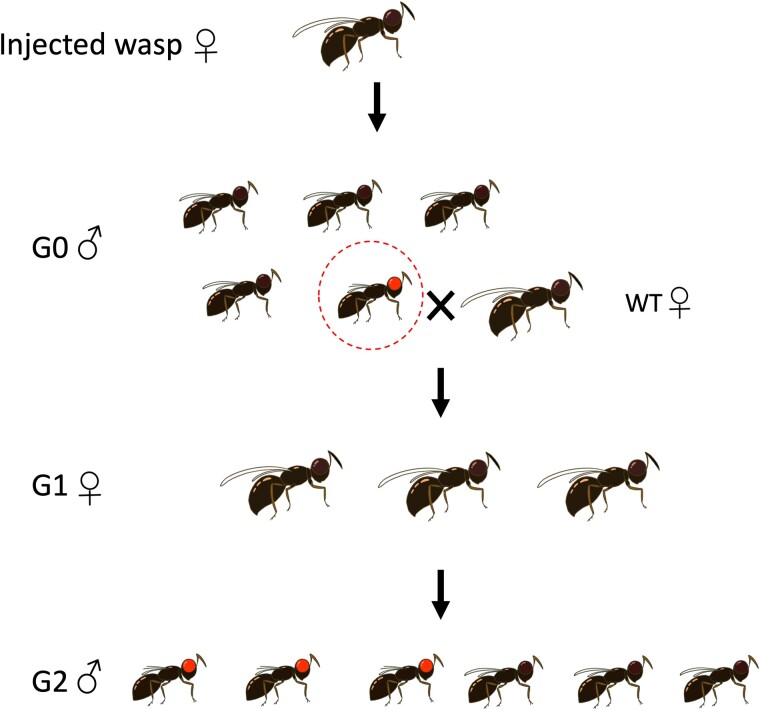
Genetic crossing scheme to test the heritability of the G0 red-eyed phenotypes. A given G0 mutant male is crossed with a WT virgin female to produce G1 generation progeny. Unmated, heterozygous G1 females produce G2 progeny, which exhibit a ∼1:1 ratio of mutant (red-eyed) and WT (black-eyed) individuals.

Upon emergence, G0 wasps were sorted under the dissecting microscope to screen for mutant eye pigmentation. Wasps that exhibited mutant red eyes were collected and placed individually into glass vials (Li *et al.* 2017). Each G0 mutant was crossed with a WT female to test for heritability of the mutant trait. Following mating for 2 days, each G0 mutant male was preserved at −20°C for subsequent DNA extraction and sequencing of the targeted allele (Fig. 3).

Prior to the emergence of G1 wasps, female pupae were sorted and kept in a separate vial for emergence. Since females are heterozygotes, they exhibited the WT (dark) eye pigmentation phenotype. G1 females were randomly selected and grouped in sets of 5 females per vial among 2–4 vials. Each vial received 3 hosts to allow the females to lay unfertilized G2 eggs. The resulting G2 haploid male wasps were subsequently screened for mutant eye pigmentation. A heritable eye color mutation transmitted by a heterozygous female was expected to yield approximately half of G2 males having red eyes.

### DNA sequencing of G0 mutants

To assess the nature of each induced mutation in the *cn* and *v* genes, the preserved mutant males were subjected to DNA extraction using the DNeasy Blood & Tissue Kit (QIAGEN, Germany) following the manufacturer's protocol. The targeted regions of each gene were amplified via PCR using specific primers (Table 1: Nvcn_F&R for *cn*; Nvv_R2_F&R for *v*). PCR products were visualized on a 1% agarose gel and purified using the QIAquick PCR Purification kit (QIAGEN, Germany) or the Zymoclean Gel DNA Recovery Kit (Zymo Research, USA) following the manufacturers’ protocols. Purified PCR products were sequenced by Primordium Lab (USA). Induced lesions were identified by comparison of sequences from mutant individuals to those from WT individuals.

### Conducting reverse transcription–quantitative polymerase chain reaction to measure *cn* expression

To measure expression of the *cn* gene in mutants displaying a heritable, red-eyed phenotype but lacking lesions in the *cn* gene, 3 pairs of red-eyed and WT G2 males of similar size at the dark-thorax-yellow-abdomen pupal stage were selected for RNA extraction and cDNA synthesis. cDNA was diluted 3-fold before use in quantitative polymerase chain reaction (qPCR). qPCR was performed using PowerUP SYBR Green Master Mix (Thermo Fisher Scientific, USA) on a CFX Opus 96 system (Bio-Rad, USA). Each reaction of 10 ⎧l total volume contained 5 ⎧l of SYBR Green Master Mix, 3 ⎧l of water, 1 ⎧l of diluted cDNA and 0.5 ⎧l of each primer [Table 1: qcnm_F&R for *cn*, Elongation factor 1 (EF1) 〈_F&R for *EF1*〈, and Ak3_F&R for *adenylate kinase 3* (*Ak3*)]. 〈*EF1*〈 and *AK3* were used as reference genes in qPCR (Dalla Benetta *et al.* 2019). The qPCR protocol involved an initial step of 2 min at 50°C, then 2 min at 95°C, followed by 40 cycles of 15 s at 95°C, 15 s at 55°C, and 1 min at 70°C. The relative fold change of *cn* gene expression was calculated using the delta–delta Ct method.

## Results

### DIPA-CRISPR editing of the *N. vitripennis cn* gene

To assess the potential of DIPA-CRISPR in *N. vitripennis*, we targeted the *cn* gene since it is known to be editable through other CRISPR-based approaches (Li *et al.* 2017; Chaverra-Rodriguez *et al.* 2020). We varied 2 injection parameters: The concentration of Cas9–sgRNA reagents and the injection volume. Injections were conducted on adult females 0–45 h postemergence, and all G0 mutant progeny reported below were produced only from females injected at 3–31 h postemergence.

For the condition consisting of the higher reagent concentration with a larger injection volume, a total of 117 virgin females were injected (Table 2). Two of these females produced 4 G0 male mutants that exhibited a distinctive, bright red-eyed mutant phenotype (Fig. 1). The total number of G0 progeny resulting from this trial was not recorded given that it was an initial step to determine if this method would produce mutants. Each mutant G0 male was crossed with a WT female to obtain G1 female progeny, which should be heterozygous for an edited allele and the WT allele (Fig. 3). Consistent with this prediction, unmated G1 females produced G2 male progeny, half of which contained fully pigmented (black) eyes and the other half which showed the red-eyed mutant phenotype, confirming that the induced mutations were heritable. DNA sequencing confirmed the presence of insertion-deletion (in-del) lesions at the sites targeted by sgRNAs (Fig. 1). The combination of the lower reagent concentration with larger injection volume was used to inject 179 unmated females (Table 2). The number of G0 male progeny produced by these females was recorded, allowing us to calculate the GEF. Out of 4,970 G0 males, 4 were mutant for eye color, resulting in a GEF of 0.08%. Subsequent DNA sequencing and genetic crosses confirmed successful editing of *cn* and the heritability of these mutant alleles, respectively.

**Table 2.** DIPA-CRISPR injection information.

Gene	Wasp stage	Injection parameter	Injected wasps	Wasps produced offspring	G0	Red-eyed mutants	Lesion confirmed	Heritability confirmed	GEF
*cn*	Adult	high conc., large volume	117	na	na	4	4	4	na
*cn*	Adult	low conc., large volume	179	82	4,970	4	4	4	0.08%
*cn*	Adult	high conc., small volume	313	157	7,271	0	0	0	0
*cn*	Adult	low conc., small volume	262*^a^*	153	9,555	3	2	3	0.03%
*cn*	Black pupa	high conc., large volume	120	43	3,124	0	0	0	0
*v*	Adult	low conc., large volume	181	159	10,435	6	6	6	0.05%

One outlier injection was excluded from this trial, which produced approximately half of G0 progeny (*n* = 36) that showed red-eyed phenotype.

We then conducted injections with the 2 different reagent concentrations combined with the smaller injection volume. With this approach, we observed notable variation in the outcome. Five hundred and seventy-five injected, unmated females produced 16,826 G0 males (Table 2). No mutants were produced by the females injected with the higher concentration of reagents. In contrast, 2 low-injected females yielded three mutant progeny. Two of them were confirmed by sequencing to have lesions, while the third progeny had a heritable mutant phenotype but no detectable lesion. Additionally, there was 1 outlier injected female that produced 36 G0 mutants, in which their mutant phenotype was heritable but there was no detectable lesion in the entire *cn* gene, including its introns. To test if *cn* expression was affected by a lesion in the transcriptional regulatory region(s), we performed reverse transcription–qPCR (RT–qPCR) to measure *cn* expression in these mutants, using *cn* expression in WT wasps as a control. No significant difference in expression was detected (Paired *t*-test, *P*-value > 0.05), suggesting that alteration of the regulatory region did not underlie their mutant phenotype. Further, this finding argued against any hindrance of *cn* expression due to epigenetic effects. These experiments suggest that the progeny of these particular mutant G0 females carry off-target lesions in another gene(s) within the ommochrome pathway that result in the same mutant red phenotype.

To test the effectiveness of DIPA-CRISPR earlier in development, we injected Cas9–sgRNAs into fully pigmented female pupae; these individuals are 1–2 days younger than the adult females used in this study. Pupae have the technical advantage that they are much easier to inject because they are motionless. In a pilot test of ∼900 injected female pupae, ∼500 emerged as adults and these individuals did not produce any G0 mutants. In a more carefully measured manner, we further injected 120 female pupae, and 43 successfully emerged into adulthood. Like in the pilot, these unmated females produced 3,124 G0 males but no mutants were identified from these individuals (Table 2).

In short, we were able to generate edits in the *cn* gene at both the higher and lower concentrations of Cas9–sgRNAs, but a larger injection volume was necessary. Moreover, while all 3 of our sgRNAs induced lesions, there was variation regarding their mutational efficiency. From the mutants generated in the *cn* gene, the CNex3 sgRNA exhibited the highest efficiency, producing lesions in 8 individuals, sgRNA3_Li produced lesions in 5 individuals, and CNex7 produced lesions in 3 individuals. Notably, any given mutant produced from these experiments contained up to 3 different lesions in *cn*, each corresponding to a site targeted by 1 of the 3 sgRNAs. In two different mutants, *cn* was edited by 2 sgRNAs targeting sites separated by ∼700 bp, thus leading to a deletion of this entire region.

### Assessing DIPA-CRISPR using a different wasp pigmentation gene

**Fig. 4.**
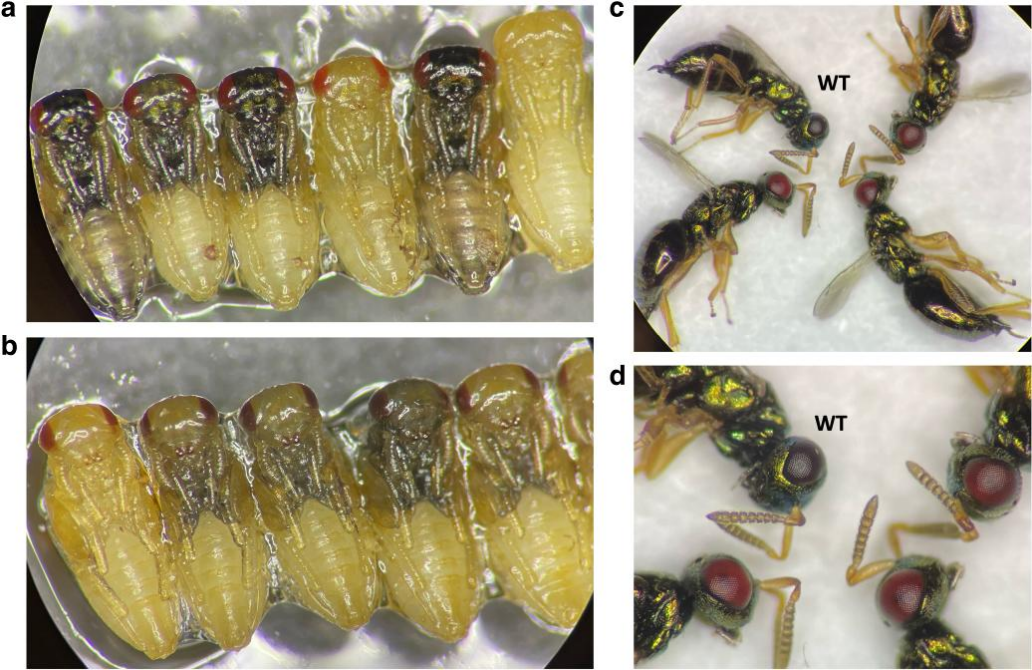
RNAi-treated *N. vitripennis*. a) dsRNA-injected pupae at the yellow-red-eyed stage and the half-yellow-half-dark stage; b) WT pupae at the yellow-red-eyed stage and the half-yellow-half-dark stage; c) WT (up-left) and RNAi-treated adult wasps; d) enlarged eye image of panel C.

So far, *cn* is the only *N. vitripennis* gene to be targeted by CRISPR. We therefore targeted another eye pigmentation gene, *vermillion* (*v*), using DIPA-CRISPR. To first assess if this gene would produce a clearly visible phenotype, we targeted it with sRNAi. A total of 14 female and 88 male pupae were dsRNA-injected, and 92 successfully emerged into adults. These individuals exhibited an expected, red-eyed mutant phenotype (Fig. 4), although the intensity varied among these individuals, likely due to variation in *v* transcript reduction. Nevertheless, the phenotype was clearly and consistently visible, confirming the suitability of *v* as a potential DIPA-CRISPR target.

We then injected a total of 181 female adults with Cas9 and a combination of 3 sgRNAs that were designed to target the *v* gene, using the lower concentration and larger volume condition. A total of 10,435 G0 males were produced from these females, from which 6 were red-eyed (GEF = 0.05%; Fig. 2, Table 2). Further genetic crosses and DNA sequencing confirmed that the resulting mutant *v* alleles were heritable, and they harbored specific lesions that matched the sgRNA-targeted sites. Among the three sgRNAs, the Vex5 sgRNA was the most efficient, producing different *v* lesions in 4 individuals. Vex3 sgRNA produced 2 different lesions in 2 individuals, while Vex4 sgRNA produced no lesions.

## Discussion

While CRISPR-based gene-editing holds strong potential for reverse-genetic-based gene studies, its application in many nonmodel insects has been limited by the challenges of reagent delivery. In this study, we induced gene lesions in *N. vitripennis* through DIPA-CRISPR, expanding the scope of insects for which this relatively facile method has been successfully applied. Two different eye pigmentation genes were successfully targeted, helping to demonstrate the applicability of this method at different targeted regions in the wasp's genome. As expected, the types of in-del lesions induced in our study were like those produced using other CRISPR-based delivery methods in *N. vitripennis* (Li *et al.* 2017; Chaverra-Rodriguez *et al.* 2020). Of the two different reagent concentrations and injection volumes tested on adult wasps in our study, the larger volume with either concentration yielded mutations more consistently than the smaller volume: The larger volume injection induced lesions at an infrequent but consistent rate that were confirmed by both allele sequences and genetic crosses. This effect may be due to a greater bulk of solvent delivered with the larger injection volume, which could more thoroughly inundate the wasp's tissues with reagents during injection.

Two of the injected wasps, which were treated with the low concentration and small volume of reagents, produced red-eyed progeny that harbored no in-del lesions at the intended target site or elsewhere in the gene. Thus, we conclude that these mutants are likely to be the result of off-target effects on other ommochrome-pathway genes. A similar off-target mutant was detected in a previous study using ReMOT (Chaverra-Rodriguez *et al.* 2020), albeit with a brown-eyed phenotype rather than the red-eyed phenotype observed in our study. No other unintended mutant effects were observed in our experiments, although any subtle phenotypes may have been missed. The occurrence of off-target mutant effects in nontarget genes producing the same or a similar phenotype being screened for underscores the importance of validating each mutant through PCR-based sequencing. Additionally, such off-target mutants may be of interest in studies aimed at discerning genetic interactions within cellular pathways of interest.

Despite the ease of performing DIPA-CRISPR in *N. vitripennis*, one notable caveat to this approach is the overall low mutation efficiency when compared to other tested insects. Specifically, the GEF value for the wasp *cn* gene was 0.08%, compared to values observed in cockroaches, mosquitoes, and the red flour beetle, which ranged from 3.5 to 71.4% (Shirai *et al.* 2022, 2023). The mutation efficiency was similarly low for the wasp's *v* gene (GEF = 0.05%). These patterns raise an important question: What factors underlie this low editing efficiency in *N. vitripennis* and, more broadly, the wide range in DIPA-CRISPR efficiency among different insects?

Part of the answer may be explained by species-specific differences in molecular import during vitellogenesis, a phase of egg development typified by rapid yolk protein production and egg cytoplasmic growth. In some insects such as cockroaches and mosquitoes, yolk protein precursors are synthesized primarily in the fat body of females and subsequently secreted into the hemolymph (Valle 1993). From there, these proteins are taken up by the developing oocytes through receptor-mediated endocytosis (Valle 1993; Tufail and Takeda 2009). Previous in vitro studies suggest that, in certain insects, oocytes not only selectively uptake yolk proteins but also large proteins such as mouse IgG and γ-globulin nonselectively (Kindle *et al.* 1988; Koller *et al.* 1989). DIPA-CRISPR experiments in the cockroach and the red flour beetle employed un-tagged Cas9, thus relying on nonspecific uptake of RNPs during vitellogenesis (Shirai *et al.* 2022). In contrast to the high mutation rates observed in the cockroach and red flour beetle, attempts to use DIPA-CRISPR in the fruit fly, *D. melanogaster*, yielded no mutations (Shirai *et al.* 2022). In *D. melanogaster*, vitellogenins are synthesized in both the ovary and the fat body (Brennan *et al.* 1982), and the patency window, during which intercellular spaces between follicle cells open up for hemolymph proteins to enter the oocyte, is short (Zimowska *et al.* 1994; Isasti-Sanchez *et al.* 2021), therefore leading to a much-reduced rate of yolk protein uptake from outside the ovary (Shirai *et al.* 2022). Based on the high mutational rates achieved by DIPA-CRISPR in the cockroach and red flour beetle, it is likely that these insects have vitellogenic import systems that are both highly efficient and permissible to nonspecific protein uptake. Our injection results therefore suggest that *N. vitripennis* would be intermediary in vitellogenic molecular uptake between the red flour beetle and the fruit fly.

Another factor that may influence DIPA-CRISPR efficiency is the specific mode of oogenesis. Insects including the jewel wasp and the red flour beetle (Parthasarathy *et al.* 2010; Shirai *et al.* 2022) have continuous oogenesis, in which the ovary produces developing eggs continually in an assembly line throughout adulthood. In contrast, other insects like the cockroach and mosquitoes produce eggs discontinuously (Hagedorn 1974; Pascual *et al.* 1992; Shirai *et al.* 2022, 2023); in this mode, most or all eggs produced by a given female develop together in a single batch. Thus, insects with continuous oogenesis will, at the time of injection, have only a small portion of developing eggs that are undergoing vitellogenesis, ensuring that at least some eggs will import CRISPR RNPs before these reagents are degraded. Reciprocally, insects with discontinuous oogenesis have the potential for targeting many eggs at once if injection corresponds to the specific window of time involving vitellogenesis. However, if this window is missed, few or no mutations would result. The low mutational frequency observed here using DIPA in *N. vitripennis* may stem from a low level of RNP import into the few eggs that are undergoing vitellogenesis following injection. Additionally, the lack of mutations produced by females injected during the late pupal stage may reflect a paucity of vitellogenic eggs at that time, a conclusion supported by previous observations (Dong *et al.* 2008). The abundance of vitellogenin in the wasp's hemolymph peaks at ∼6 h postemergence, while ovarian vitellin reaches its peak at ∼24 h postemergence in *N. vitripennis* (Dong *et al.* 2008). These patterns suggest that the uptake of vitellogenin from hemolymph is elevated between 6 and 24 h postemergence. In our study, the wasps that produced mutants were injected at 3–31 h postemergence, aligning more or less with the timing reported in Dong *et al.* (2008). However, timing the injection of CRISPR reagents to correspond exactly with the period of largest abundance of vitellogenic eggs in *N. vitripennis* will require further investigation.

In summary, each CRISPR delivery method—DIPA, ReMOT, and direct embryo microinjection—has both merit and drawback, and the decision of when to use DIPA over the other methods may depend on several considerations. Embryo microinjection is by far the most efficient CRISPR approach in *N. vitripennis* but performing microinjection into early wasp embryos involves technical challenges and may not be feasible for all *Nasonia* labs. Regarding ease of CRISPR reagent delivery, DIPA and ReMOT are equally straightforward, involving injection into pupae or adults; while pupae are immobile, adult movement can be greatly reduced if the injections are performed on ice. In contrast, injection into embryos involves substantial challenges in achieving a high embryonic survival rate due to the small size and fragile nature of embryos. Additionally, injected embryos must be carefully transplanted into prestung flesh fly hosts for subsequent development. Between DIPA and ReMOT, mutational efficiency may be moderately higher (GEF = 0.24%) using the latter approach, given that vitellogenic RNP import is enhanced by the P2C–tagged Cas9. However, the production of purified P2C–Cas9 is laborious and outside the ability of some labs, and, alternatively, it may be difficult to procure from other research groups. Moreover, it is likely that a wasp-specific P2C fusion protein is preferred, although there is evidence that the use of P2C from *D. melanogaster* is functional as an enhancer of RNP uptake in *N. vitripennis*, albeit at a reduced rate (Chaverra-Rodriguez *et al.* 2020). Other helper reagents, such as Branched Amphipathic Peptide Capsules, have been tested previously as potential uptake mediators but they did not yield GEF values substantially higher than ours achieved here with DIPA (Chaverra-Rodriguez *et al.* 2020). Ultimately, DIPA in *N. vitripennis* may be best suited for the generation of infrequent mutant alleles resulting in highly visible phenotypes that are easily spotted from among numerous WT sibling progeny. Given the low mutation efficiency observed here using DIPA-CRISPR, its usefulness in generating loss-of-function alleles in genes that do not present visibly-distinctive phenotypes will be limited until a higher mutation efficiency is achieved or an affordable method for screening mutations is developed. Finally, it remains to be determined whether direct injection of CRISPR reagents into females can be adapted for achieving integration of exogenous DNA. Such a feat will require the development of means for efficient delivery and uptake of donor DNA by vitellogenic eggs along with Cas9 and sgRNAs. Until then, such an aim will likely be limited to other approaches such as embryo microinjection.

## Data Availability

The authors affirm that all data necessary for deriving the conclusions of this article are present within the text, the data tables, and the figures.
